# Genomic and functional characterization of a lytic *Klebsiella* phage UHKP with antibiofilm activity

**DOI:** 10.3389/fmicb.2026.1775638

**Published:** 2026-03-02

**Authors:** Muhammad Hassan, Iqbal Ahmad Alvi, Sadiq Noor Khan, Dawood Ahmed, Muhammad Asif, Afshan Saleem

**Affiliations:** 1 Department of Medical Lab Technology, The University of Haripur, Haripur, Khyber Pakhtunkhwa, Pakistan; 2 Department of Microbiology, Afghan International Islamic University, Kabul, Afghanistan; 3 Department of Microbiology, Hazara University Mansehra, Mansehra, Khyber Pakhtunkhwa, Pakistan; 4 Department of Microbiology, Government College University Lahore, Lahore, Punjab, Pakistan; 5 Department of Microbiology, The University of Haripur, Haripur, Khyber Pakhtunkhwa, Pakistan

**Keywords:** bacteriophage therapy, biofilm disruption, genomic characterization, *Klebsiella pneumoniae*, multidrug resistance, UHKP

## Abstract

*Klebsiella pneumoniae* is an opportunistic pathogen causing severe hospital-acquired infections, and it rapidly acquires multidrug resistance. Its robust biofilm formation further complicates treatment and drives interest in phage therapy. A phage UHKP was isolated from hospital sewage using an MDR *K. pneumoniae* strain (*KP-*03). UHKP formed clear plaques and having phage titer 2.3 × 10^9^ PFU/mL. Host-range testing on 19 clinical isolates showed a narrow spectrum. Only four MDR strains (*KP-*03, *KP-*05, *KP-*08, *KP-*11) and one K-17 serotype were lysed, with no activity on other strains or species. One-step growth analysis yielded a 30 min latent period and 85 PFU burst size. In planktonic culture, UHKP at MOI 1 stopped bacterial growth by 4 h and cleared cultures by 8 h, whereas at MOI 0.1 killing was delayed and incomplete. In static biofilm assays, UHKP eradicated 98% of 24-h and 96% of 48-h biofilm biomass by 24 h (MOI 1). Clearance of 72 – 96 h biofilms was limited (≤ 87% by 24 h). UHKP possesses an icosahedral head of 56 ± 3 nm and a short, non-contractile tail measuring around 15 ± 2 nm. Genome sequencing revealed a 62,542 bp dsDNA genome (56.6% GC) encoding 77 ORFs, and phylogenetic analysis placed UHKP in the genus *Lastavirus*. UHKP carries no lysogeny, toxin or antibiotic-resistance genes.

## 1 Background

*Klebsiella pneumoniae* is an extremely adaptable pathogen, playing a primary role in opportunistic healthcare-associated infections, and being relevant also in some community-acquired infections. It exhibits high tendency for developing resistance to antibiotics, including carbapenems, and contribute to the major carbapenem-resistant *Enterobacteriaceae* worldwide (Di Pilato et al., 2024). Globally, it is responsible for more than 69,000 deaths annually, thereby poses a major public health threat (Mestrovic et al., 2022). It is now responsible for elevated bacterial infections in the European region currently, being highly prevalent in nosocomial infections (Cassini et al., 2019). While it commensally inhabits the human gastrointestinal tract, it can cause severe complications in critical and immunosuppressed individuals such as septicemia, surgical site or wound infections, urinary tract infections and pneumonia (Ferriol-González et al., 2024).

The global rise in antibiotic resistant pathogens has caused a severe public health threat. These antibiotic resistant pathogens can result in increased mortality and morbidity, that extends hospital stays and elevates treatment expenses compared to other pathogens. Because of the antimicrobial resistance, *K. pneumoniae* is a global public health concern and has been included in ESKAPE group that is known for high virulence and remarkable resistance (Asif et al., 2023).

*K. pneumoniae* is associated with hospital acquired infections having *in vivo* and *in vitro* potential of forming biofilms significantly in individuals possessing medical devices including urine catheters, artificial and dental implants and endotracheal tubes. The biofilm lifecycle progresses through distinct phases including initial surface attachment followed by microcolony development, structural maturation, and eventual release of planktonic cells (Devanga Ragupathi et al., 2024). Bacterial biofilms exhibit dramatically enhanced antibiotic resistance compared to their planktonic bacteria, with the resistance reported to be 1,000-fold greater. The protective biofilm matrix also effectively shields bacteria from immune clearance mechanisms like phagocytosis (Zheng et al., 2018). With 60–80% microbial infections now comprising biofilms, treatment options are limited, particularly for *K. pneumoniae* biofilms that demonstrate nearly complete resistance to first-line antibiotics. This resistance makes biofilm eradication significantly more difficult than treating planktonic bacterial population (Li et al., 2024).

The accelerating rise in antimicrobial resistance is now responsible for approximately 2.8 million resistant infections annually alone in the U.S., with these cases demonstrating worst clinical outcomes showing increased morbidity, mortality, and treatment costs. Current estimates attribute 35,000 annual deaths associated with such resistant infections (Ullah et al., 2024). A particularly alarming aspect is the development of resistance across all classes of beta-lactam antibiotics, ranging from basic penicillins to advanced carbapenems and even colistin, which is often considered the last resort treatment for multidrug-resistant infections. Bacterial pathogens have developed multiple mechanisms to escape antibiotic activity. These include modifying their porin channels to prevent drug entry, overproducing efflux pumps to remove antibiotics from cells, altering drug targets through genetic mutations, forming protective biofilms, and producing enzymes that chemically modify and inactivate antibiotics. Each resistance strategy particularly poses unique challenges for clinical treatment and complicates infection management. The rapid emergence of these mechanisms following antibiotic exposure highlights the remarkable adaptability of these pathogens and highlights the urgent need for novel therapeutic alternatives (Sundaramoorthy et al., 2022). Current treatment protocols for bacterial infections primarily rely on antibiotic administration, whether as monotherapy or in combination. However, the therapeutic approach is declining as the sensitivity of effective antibiotics continuously decreases. This concern is pushing us closer to a potential post-penicillin era where common infections may become untreatable. Examination of the Clinical and Laboratory Standards Institute (CLSI) guidelines shows a disturbing pattern where numerous antibiotics previously recommended for these pathogens have been removed from treatment protocols since 2010, while only a limited number of new antibiotics have been introduced. The emerging reports of resistance developing against several of these recently approved antibiotics is the major concern that further limits the available treatment options (Mulani et al., 2019).

The scarce rate of novel antibiotic development has created an urgent need to explore complementary approaches for managing severe bacterial infections (Alvi et al., 2021). Currently, multiple innovative therapeutic strategies, including combination antibiotic regimens with resistance-modifying adjuvants, naturally occurring antimicrobial peptides, light-activated photodynamic treatments, engineered antibody therapies, plant-derived bioactive compounds, nanoscale antimicrobial materials and targeted bacteriophage applications are being tested (Mandal et al., 2014; Kaur, 2016). Among these strategies, bacteriophage therapy emerges as one of the most potent options against antibiotic resistant pathogenic bacteria (Khan et al., 2023). Bacteriophage or phage therapy has gained attention as a potential solution to the global antibiotic resistance crisis. These specialized viruses infect and lyse specific bacterial strains, offering a targeted approach against pathogenic bacteria. Current clinical investigations are evaluating phage applications for diverse infections, including those involving treatment-resistant biofilms that often withstand conventional antibiotics. Unlike broad-spectrum antibiotics, phage therapy demonstrates precise specificity, selectively eliminating harmful bacteria while minimally disrupting commensal microbiota. Phage therapy presents additional advantages as a sustainable treatment alternative, given the self-replicating nature of phages at infection sites (Atique et al., 2024). A major advantage of phage therapy lies in their natural host specificity and ability to replicate at infection sites, enabling targeted bacterial elimination (Alvi et al., 2021). Phages exert their antimicrobial effects through direct interaction with bacterial cells, resulting in biofilm destabilization through enzymatic breakdown of extracellular matrices, complete lysis of pathogenic cells, and subsequent clearance of infection (Shafique et al., 2017).

This study aims at isolating and characterizing a lytic phage UHKP, active against a clinical isolate of multidrug-resistant *K. pneumoniae*. Comprehensive evaluation was conducted to determine its therapeutic suitability, including plaque morphology, host range, multiplicity of infection, planktonic growth inhibition, and biofilm disruption across different maturation stages. UHKP was further assessed for stability under varied pH, temperature, and storage conditions, along with one-step growth parameters such as latent period and burst size. Complete genome sequencing and annotation, absence of virulence or resistance determinants established its strict lytic nature. This study provides detailed evidence that UHKP combines effective anti-biofilm activity with genomic safety, supporting its potential as a promising candidate for phage therapy against Multidrug-resistant (MDR) *K. pneumoniae*.

## 2 Materials and methods

### 2.1 Laboratory setup

All pathogens were handled in a BSL-2 level laboratory in biosafety cabinet type 2, keeping in view strict compliance with basic microbiological techniques.

### 2.2 Bacterial strain isolation and characterization

Clinical samples from wound exudate, bacteremia, pus, and urinary tract infections, processed at the Department of Pathology, Ayub Medical Complex Abbottabad, were used for bacterial isolation using routine culture techniques. Isolates were characterized biochemically through API 20E and 20NE identification panels. For long-term storage, bacterial stocks were preserved in 25% glycerol solution at −80 °C. Log phase cultures were propagated in Luria-Bertani (LB) medium, with both broth and solid agar for maintenance. Antimicrobial susceptibility patterns were determined by the Kirby-Bauer disk diffusion assay on Mueller Hinton agar according to the CLSI guidelines.

### 2.3 Bacteriophage isolation, purification, and quantification

Environmental samples from hospital sewage in the Hazara region of Khyber Pakhtunkhwa (KPK) were screened for phages using *K. pneumoniae* strain 03 (*KP-*03) as the primary host organism. Initial detection of bacteriophage activity was performed using a spot assay, in which filtered phage lysates were spotted onto bacterial lawns to observe zones of lysis indicative of phage presence. Quantitative analysis of phage infectivity was subsequently carried out using the double-layer agar, where phage suspensions were mixed with host bacteria and overlaid onto agar plates to enumerate distinct plaques representing individual infectious phage particles (Asif et al., 2023). The mean titer of phage was determined from three biologically independent plaque assays. Purified phage was stored in LB broth at 4 °C for short-term use, whereas, for long-term archival storage, phage stocks were supplemented with 25% glycerol and maintained at −20 °C and −80 °C.

### 2.4 Multiplicity of infection (MOI) optimization

To establish the phage-to-bacteria ratio (MOI) yielding maximal viral output, logarithmic-phase *KP-*03 cultures [(10^9^ colony forming units (CFU/mL))] were inoculated with UHKP phage lysate at MOI 1, and 0.1. Infection kinetics were monitored during 24-h incubation at 37 °C under aerobic shaking conditions (150 rpm). Post-infection titers were quantified using the standardized double-layer agar assay to identify the MOI optimizing phage propagation efficiency.

### 2.5 Host range assessment

The host specificity of UHKP phage was determined by spot testing against 19 clinical *K. pneumoniae* isolates. The host range of the bacteriophages was assessed using two standard techniques: the spot assay as described by Kutter (2009) and the double-layer agar overlay method following (Mocé-Llivina et al., 2004). Bacterial strains that showed a positive response in the spot test were subsequently used to evaluate the efficiency of plating (EOP). For this, equal concentrations of phage suspensions were applied to both the original host and the test strains, and the resulting phage titers were determined according to Kutter (2009). The EOP was calculated using the following equation:


$$\begin{array}{l} \text{EOP}=\\ \;\left(\text{Number of plaques on test strain}\right)/(\text{Number of plaques }\\ \;\text{on original host strain}) \end{array}$$

Cross-species infectivity screening included non-reference clinical strains (collaboratively sourced from the Microbiology Laboratory, Abbottabad Medical Complex) and a panel of clinically relevant pathogens: *Escherichia coli* (*n* = 6), *Enterobacter* spp. (*n* = 4), *Pseudomonas* spp. (*n* = 6), and *Staphylococcus aureus* (*n* = 5).

### 2.6 Assessment of growth reduction against planktonic cultures

The antibacterial activity of UHKP phage against planktonic cultures was quantified using a standardized growth inhibition assay. Exponentially growing bacterial suspensions (1.64 × 10^8^ CFU/mL in LB broth) were co-incubated with phage lysate (1.64 × 10^7^ plaque forming units (PFU/mL) and 1.64 × 10^8^ PFU/mL) at MOI of 0.1 and 1, respectively. Control groups included bacteria only (positive control) and LB medium (negative control). All experimental and control flasks were subjected to aerobic growth conditions (37 °C, 150 rpm agitation) over a 12-h period, with bacterial density monitored via optical density measurements (OD_600_) at the intervals of 2-h for up to 12 h.

### 2.7 Biofilm development kinetics of host strain

The biofilm-forming capacity of the *KP-*03 strain was evaluated in polystyrene microtiter plates using a dual analytical approach: crystal violet (CV) staining for biomass quantification and viable cell enumeration methodologies (Tabassum et al., 2018; Haney et al., 2021). Biofilm dynamics were monitored over a 6-days maturation period, with daily CFU counts and OD_600_ measurements analyzed temporally to map developmental progress. CFU datasets were subsequently leveraged to estimate phage MOI parameters for optimized biofilm targeted therapeutic interventions.

### 2.8 Biofilm disruption potential of UHKP in microtiter plate assays

The biofilm therapeutic strategy was developed following established phage treatment protocol (Abedon et al., 2021). Mature *KP-*03 biofilms (1–4 days) were subjected to three sequential washes with sterile physiological saline (0.85% NaCl) to remove loosely attached cells, followed by resuspension in 100 μL of LB broth to sustain residual metabolic activity. Empirical MOI determination relied on age-stratified bacterial loads quantified in CFUs via viable cell counts: 2 × 10^4^ CFU (24 h), 1.79 × 10^6^ CFU (48 h), 2.0 × 10^9^ CFU (72 h), and 1.575 × 10^9^ CFU (96 h). To standardize therapeutic dosing, UHKP lysate (1.64 × 10^9^ PFU/mL in SM buffer) was volumetrically titrated to achieve MOI values of 1 and 0.1 relative to biofilm-embedded bacterial densities. Final reaction volumes were adjusted to 200 μL across all experimental and control wells to ensure experimental consistency. Sterile saline was used to equalize liquid volumes, when required. Experimental controls comprised duplicate wells containing untreated biofilms (positive controls) and sterile LB broth (negative controls). Positive control wells received sterile SM buffer as a mock treatment substitute for phage lysate to account for potential buffer interference. Viable bacterial counts were quantified at baseline (0 h) and following 6, 12, and 24 h of therapeutic intervention for both treated and untreated groups. Antimicrobial efficacy was evaluated through two metrics: (1) Log reduction in CFUs/mL was calculated using the formula Log reduction = log_10_ (T/Z), where T represents bacterial CFUs/mL in treated samples and Z corresponds to CFUs/mL at the zero-time point or untreated biofilm controls; and (2) Percentage reduction in CFUs/mL, determined by the equation percentage reduction = (1 - T/U) × 100, where T denotes bacterial CFUs/mL in treated samples and U signifies CFUs/mL in untreated controls. For microscopic visualization of biofilm architecture, adhered microbial communities were subjected to crystal violet staining following protocols adapted from Haney et al. (2021). High-resolution imaging was performed using an IRMECO IM-200 inverted light microscope system (Germany) equipped with 20X magnification capabilities, enabling detailed structural analysis of the stained biofilm matrices.

### 2.9 Environmental stability profiling (pH/temperature)

The environmental stability of UHKP phage (1.67 × 10^9^ PFU/mL) was systematically evaluated through thermal and pH tolerance assays using an already reported method (Alvi et al., 2020). Phage suspensions underwent 1- and 2-h exposures to acidic-to-alkaline conditions (pH 3–10) and thermal stress at 37 °C, 45 °C, 60 °C, and 80 °C, with post-treatment viability quantified via plaque assays. For preservation stability, phage stocks were maintained at four temperature regimes (25 °C, 4 °C, −20 °C, and −80 °C) over 1, 6, and 12 month intervals, with phage quantification done periodically. Statistical comparisons between experimental groups were conducted using one-way ANOVA, supplemented by Tukey's *post hoc* test for multiple analysis.

### 2.10 Latent time and burst size determination

Mid-logarithmic phase bacterial cultures (1 × 108 CFU/mL) were harvested via centrifugation (2000 × g, 5 min) and resuspended in 500 μL nutrient broth. Adsorption kinetics were initiated by supplementing the suspension with UHKP phage lysate at MOI 0.1, followed by a 1-min incubation at 37 °C. To separate phage-bound bacteria, brief high-speed centrifugation (10,000 × g, 30 s) was done and pelleted cells were obtained, discarding the supernatant. The pellet was transferred to fresh 100 mL LB broth and incubated at 37 °C. Aliquots (1 mL) were collected at 5-min intervals over 60 min. The aliquots were immediately centrifuged (10,000 × g, 30 s), with phage titers quantified from supernatant via plaque assay. Burst size calculations were derived from the ratio of progeny phage yield to initial adsorbed phage population.

### 2.11 Transmission electron microscope (TEM) of the phage

Structural characterization of UHKP phage was conducted via transmission electron microscopy (TEM) at the University of Leicester, UK. Phage suspensions (10 μL) were adsorbed onto glow-discharged 200-mesh Formvar/carbon-coated copper grids for 2 min. Excessive liquid was removed via capillary action using filter paper, followed by two sequential washes with ultrapure water (10 μL, 30 s each). Phages were stained by applying 10 μL of 1% (w/v) uranyl acetate for 30 s, after which grids were air-dried under ambient conditions. Imaging was performed using a JEM-1400 TEM system (JEOL UK) operated at 100 kV accelerating voltage. The micrographs were reviewed and calibrated using ImageJ (NIH) software (Asif et al., 2023).

### 2.12 Bacteriophage whole-genome sequencing

Phage genome was isolated from PEG/NaCl precipitated phage concentrates utilizing a commercial nucleic acid purification kit (Norgen Biotek, Cat. #46850), with DNA concentration determined via spectrophotometric analysis (NanoDrop™). To confirm nucleic acid composition, isolated DNA was enzymatically digested with DNase I and RNase A, while thermos Table S1 nuclease assays differentiated double stranded vs. single-stranded DNA configurations. Whole genome sequencing was performed at Macrogen Korea (Illumina NovaSeq 6000, 2 × 150 bp paired-end sequencing). Raw sequence data was quality filtered using Trimmomatic (sliding window: 4:20; MINLEN: 50), followed by k-mer frequency distribution analysis to evaluate genomic complexity. High fidelity reads were *de novo* assembled into a single contiguous sequence using SPAdes v3.15.5 (Bankevich et al., 2012).

### 2.13 Functional genome annotation

Open reading frames (ORFs) were computationally predicted using GeneMarkS (Besemer et al., 2001) and the RAST annotation server (Overbeek et al., 2014). Predicted ORF validity was corroborated by identifying Shine-Dalgarno ribosomal binding sites via the PECAAN algorithm. Functional annotation of putative proteins employed a multi-tiered bioinformatics workflow: structural domain identification was performed using InterProScan, NCBI blastp, RAST (Aziz et al., 2008), Pfam (Mistry et al., 2021) https://www.ebi.ac.uk/interpro/), and CATH (https://www.cathdb.info/.To) determine the protein domains and families, as well as to assign putative functions to the predicted ORFs, NCBI BLASTp (https://blast.ncbi.nlm.nih.gov/), HHpred (Zimmermann et al., 2018) (https://toolkit.tuebingen.mpg.de/tools/hhpred), and UniProtKB were collectively utilized. Secretory signal peptides and transmembrane helices were predicted with SignalP 6.0 (Petersen et al., 2011) (https://services.healthtech.dtu.dk/service.php?SignalP) and TMHMM 2.0 (Krogh et al., 2001) (https://services.healthtech.dtu.dk/service.php?TMHMM), respectively. Rho-independent transcription terminators were identified using ARNold (Naville et al., 2011). All workflows incorporated HMMER (Potter et al., 2018) (http://www.ebi.ac.uk/Tools/hmmer/) for hidden Markov model-based sequence profiling. Transfer RNA (tRNA) screening was conducted using Aragorn v1.2 (Laslett and Canback, 2004) and tRNAScan-SE 2.0 (Lowe and Chan, 2016), with default parameters for prokaryotic genomes. Conserved transcriptional regulatory motifs were profiled via the PHIRE algorithm (Lavigne et al., 2004). Promoter sequence prediction was executed using the PhagePromoter tool by Galaxy (Sampaio et al., 2019). Codon bias was quantified through the Codon Usage Database, part of the Sequence Manipulation Suite. Genomic repeat architecture was mapped through complementary approaches. Interspersed repetitive elements were annotated with RepeatMasker, while tandem repeats were identified via Tandem Repeat Finder (Benson, 1999) (https://tandem.bu.edu/trf/trf.html). CRISPR-Cas system components were screened using CRISPRCas Finder (Couvin et al., 2018). Putative type-III secretion in phage genome were predicted through EffectorP 3.0 (Eichinger et al., 2016) (https://effectorp.csiro.au/), hosted on the EffectiveDB platform.

### 2.14 Prediction of the safety and Lifestyle of phage

The UHKP phage's life cycle was characterized through comparative analysis of three bioinformatic platforms targeting prophage regions within its genome. Intact prophage loci were mapped using PHASTER (Arndt et al., 2016), while lytic vs. lysogenic tendencies were assessed through PHACTS (McNair et al., 2012) and PhageAI (Tynecki et al., 2020). The presence of repressor or integrase gene was manually analyzed in UHKP's genome. Antimicrobial resistance gene screening was performed via the Comprehensive Antibiotic Resistance Database (CARD) (Alcock et al., 2020), utilizing its integrated BLAST and Resistant Gene Identifier (RGI) algorithms for homology-based detection of horizontally acquired resistance genes in the phage genome. Genomic screening for horizontally acquired virulence genes was performed using virulenceFinder 2.0 (Joensen et al., 2014) and cross-referenced against the Virulence Factor Database (VFDB) (Liu et al., 2019). Integrative and conjugative elements (ICEs) were profiled via ICEfinder, a computational platform for identifying mobile genetic elements mediating horizontal gene transfer. Mycotoxin biosynthesis clusters were ruled out through targeted interrogation of the genome using ToxFinder 1.0 (https://cge.food.dtu.dk/services/ToxFinder/), which screens for conserved toxin synthesis genes. Phage-host tropism validation was conducted with HostPhinder 1.1, leveraging k-mer frequency alignment to predict bacterial host specificity.

### 2.15 Evolutionary genomics and phylogenetic analysis

Phylogenetic neighbors of UHKP were identified through nucleotide homology analysis using NCBI BLASTn. Average nucleotide identity (ANI) values among top homologs were computed via the CJ Biosciences ANI calculator (Yoon et al., 2017) (http://www.ezbiocloud.net/tools/ani). Orthologous gene clustering and core-pangenome delineation were performed using CoreGene 3.5 with a BLASTP score threshold of 75%. For phylogenetic reconstruction, putative large terminase sequence was aligned to homologs retrieved from NCBI BLASTp. Phylogenetic relationships were resolved through multiple sequence alignment and tree construction in MEGA 12 (Kumar et al., 2018), employing the UPGMA algorithm with 1,000 bootstrap value. For proteome-wide evolutionary analysis, the ViPTree 2.0 server (Nishimura et al., 2017) (https://www.genome.jp/viptree/) generated a dendrogram comparing UHKP against hundreds of viral proteomes, enabling taxonomic classification through hierarchical clustering of whole-genome protein similarity. Comparative genomics further delineated proteomic divergence between UHKP and its closest homologs. Taxonomic assignment at family, genus, and species level was conducted using the VICTOR (Meier-Kolthoff and Göker, 2017) (https://ggdc.dsmz.de/victor.php), which applies the Genome-BLAST Distance Phylogeny (GBDP) method to infer phylogenies from pairwise genome alignments.

### 2.16 Phage genome packaging mechanism

To infer the phage's DNA packaging strategy, a UPGMA phylogenetic tree was reconstructed from aligned large terminase subunit sequences (TerL) of UHKP and reference phages with experimentally validated packaging mechanisms. TerL clade membership analysis provided evolutionary context for hypothesizing UHKP's packaging mode, leveraging conserved terminase motifs as molecular signatures.

### 2.17 Statistical analysis

Statistical analysis of UHKP's growth inhibitory effects was conducted in GraphPad Prism 8.0. Student's unpaired *t*-test evaluated pairwise differences in antimicrobial efficacy between treated planktonic cells/biofilms and untreated controls. One-way ANOVA assessed variance across multi-group comparisons (biofilms of varying maturation stages). *Post hoc* Tukey's multiple comparison tests resolved intergroup differences at a 95% confidence interval.

## 3 Results

### 3.1 Bacteriophage isolation against multidrug-resistant bacterial strain

The bacteriophage UHKP was isolated using a clinical MDR *K. pneumoniae* strain *KP-*03 exhibiting multi-drug resistance, as confirmed via Kirby-Bauer disk diffusion assay. Antimicrobial susceptibility testing confirmed resistance across multiple antibiotic classes, observed in all tested strains with highest resistance observed in *KP-*03, establishing this strain as a representative MDR phenotype for phage host-range characterization (Supplementary Table 1).

### 3.2 UHKP produced clear plaques with a hazy zone around

**Figure 1:**
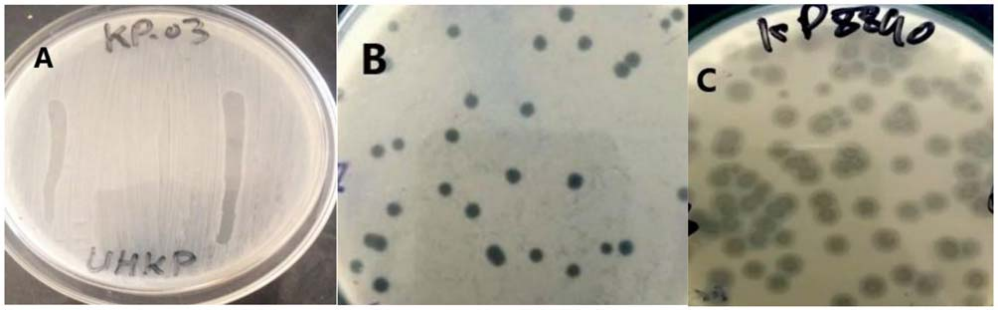
Spot assay **(A)** and plaque morphology of UHKP against *KP-*03 **(B)** and *KP-*8890 **(C)** after 24 h of incubation at 37 °C on *KP-*03 lawn.

Following a 24-h incubation period, UHKP produced well-defined, circular plaques with a clear central lytic zone ranging from 2 to 4 mm in diameter, indicating efficient phage-mediated bacterial lysis (Figure 1). Optimization assays identified a MOI of 1 as ideal for subsequent experimental workflows. Viral quantification via duplicate double-layer agar assays established a mean phage titer of 2.3 × 10^9^ PFU/mL.

### 3.3 UHKP has a narrow host range

UHKP demonstrated selective lytic activity against four *K. pneumoniae* clinical isolates (*KP-*03, *KP-*05, *KP-*08, and *KP-*11) and a confirmed K-17 serotype strain *KP-*8890 (Figure 1C) (Asif et al., 2023). No lytic effects were observed against other *K. pneumoniae* serotypes or non-*Klebsiella* species, confirming narrow host specificity (Table 1).

**Table 1:** Clinical origin, lytic activity and EOP of UHKP phage against selected *Klebsiella* strains.

**Strain ID**	**Clinical specimen**	**Isolation source**	**Lytic activity by UHKP**	**EOP**
KP−01	Urine	Hospitalized patient	–	0
KP−02	Wound swab	Hospitalized patient	–	0
KP−03	Blood	Bacteremia patient	+	1.0
KP−04	Pus	Surgical patient	–	0
KP−05	Urine	Hospitalized patient	+	0.000125
KP−06	Wound swab	Hospitalized patient	–	0
KP−07	Urine	Hospitalized patient	–	0
KP−08	Pus	Hospitalized patient	+	0.000322
KP−09	Blood	Hospitalized patient	–	0
KP−10	Urine	Hospitalized patient	–	0
KP−11	Wound swab	Hospitalized patient	+	0.000875
KP−12–KP−19	Mixed clinical specimens	Hospitalized patients	–	0
KP−8890	Clinical isolate	Reference/clinical strain	+	0.1625

### 3.4 UHKP retarded bacterial growth efficiently

**Figure 2:**
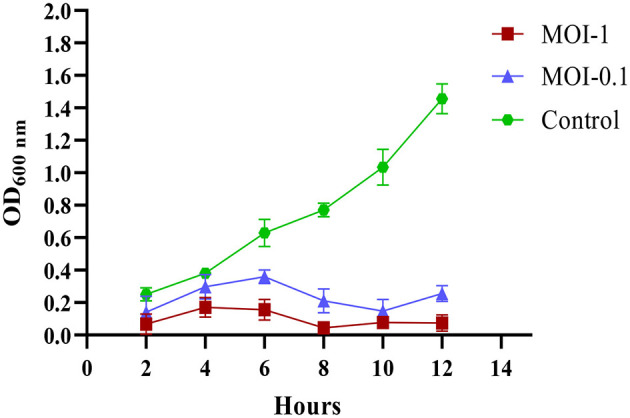
Growth reduction potential of UHKP against *K. pneumoniae* strain *KP-*03 at MOI 1 and 0.1 compared with untreated control. Error bars represent standard error of the mean (SEM).

UHKP demonstrated significant, dose-dependent inhibition of strain *KP-*03 planktonic growth over the 12-h incubation period. At MOI 1, growth suppression noticed at 4 h was compared to control, whereas inhibition increased by 12 h. The lower MOI of 0.1 showed delayed inhibition at 6 h. Complete suppression of the logarithmic growth phase was observed at MOI 1, while MOI 0.1 permitted residual bacterial growth, evidenced by final OD_600_ readings of 0.73 for MOI 0.1 compared to 0.25 for MOI 1. Visual inspection of cultures revealed complete clearance in MOI 1 flasks by 8 h. The growth curves exhibited classic bacteriophage kinetics, with an initial lag phase followed by rapid decline in optical density corresponding to host cell lysis (Figure 2).

### 3.5 Biofilm development kinetics

**Figure 3:**
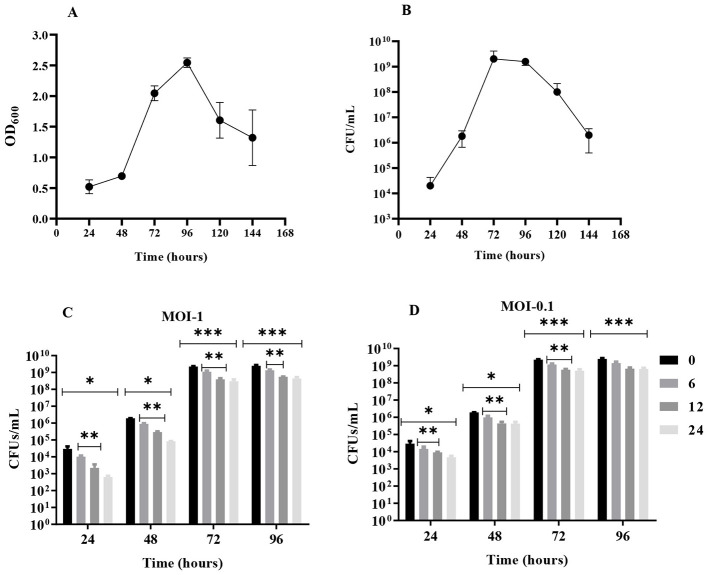
Biofilm formation and removal by UHKP. Panels A and B represent *K. pneumoniae KP-*03 biofilm development kinetics assessed by crystal violet assay **(A)** and viable count assay **(B)**. Panels C and D show biofilm removal (CFU/mL reduction) following UHKP treatment for 6, 12, and 24 h against 1–4-days-old biofilms at MOI 1 **(C)** and MOI 0.1 **(D)**, compared with baseline (*t* = 0). Bars marked with * indicate statistically significant reduction at each treatment time point relative to baseline. Bars marked with ** denote significant differences between 6– and 12-h treatments, while *** indicates significant reduction between 12– and 24-h treatments. Statistical significance was determined at *p* < 0.05.

The host strain *KP-*03 demonstrated characteristic biofilm development over the 96-h observation period. Initial adhesion (0–24 h) established microcolonies at 2.14 × 104 CFUs/well, followed by exponential growth phase (24–72 h) reaching peak density of 2.09 × 10^9^ CFUs/well. Mature biofilms (72–96 h) showed slight decline to 1.27 × 10^9^ CFUs/well, likely due to nutrient depletion and waste accumulation (Figures 3A, B).

### 3.6 UHKP efficiently removed mature biofilm

**Figure 4:**
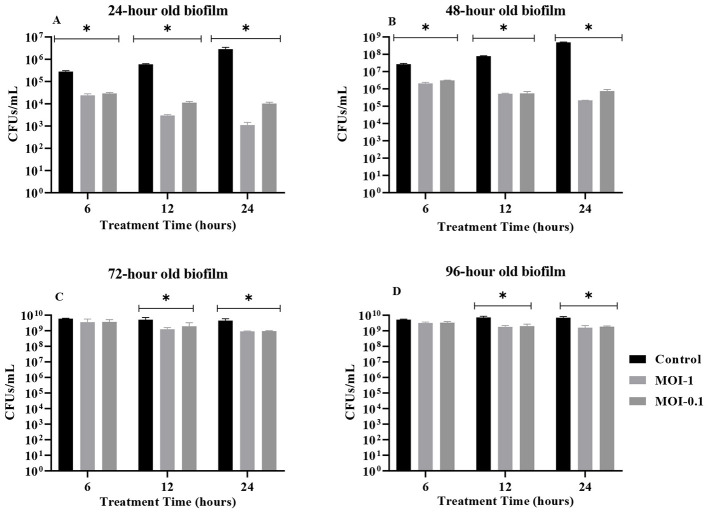
Biofilm removal (CFU/mL reduction) following UHKP treatment for 6, 12, and 24 h against 1–4 days-old *K. pneumoniae KP-*03 biofilms at MOI 1 and 0.1, compared with untreated biofilm growth control. Bars marked with * indicate statistically significant reduction relative to untreated controls (*p* < 0.05).

The anti-biofilm efficacy of UHKP was evaluated through comparative analysis of bacterial viability pre- and post-treatment, with reductions quantified relative to both baseline (*t* = 0) and untreated biofilm controls. Temporal phage activity against 24-h mature biofilms demonstrated dose-dependent eradication. MOI-1 treatment induced progressive decreases of 0.459 (65%), 1.127 (93%), and 1.665 log (98%) at 6, 12, and 24 h, respectively (Figure 3C). Increased reduction was observed in direct comparisons to untreated biofilms, with reductions of 1.071 (92%), 2.29 (99.5%), and 3.41 logs (99.96%) at 6, 12, and 24 h (Figure 4A). Statistical significance (*p* value < 0.05) was confirmed across all time points vs. controls. Mean log reductions were derived from duplicate assays, underscoring UHKP's capacity to destabilize biofilms through sustained lytic activity.

For a 48-h-old biofilm, UHKP exhibited enhanced efficacy, achieving progressive log reductions of 0.331 (53%), 0.819 (85%), and 1.365 (96%) at 6, 12, and 24 h post-treatment, when administered at MOI-1 (Figure 3C). Comparative analysis against untreated controls revealed statistically significant suppression (*p* value < 0.05) at all time points, with bacterial load reductions of 1.109 (92%), 2.176 (99.3%), and 3.354 logs (99.96%) at 6, 12, and 24 h post treatment, respectively (Figure 4B).

For 72 and 96-h-old biofilms, UHKP established diminished efficacy, with reduced clearance observed for 72– and 96-h mature biofilms. Against 72-h biofilms, MOI-1 treatment achieved log reductions of 0.305 (50%), 0.753 (82%), and 0.874 (87%) at 6, 12, and 24 h, respectively (Figure 3C). A comparable trend emerged for 96-h biofilms, where log reductions were 0.271 (46%), 0.655 (78%), and 0.748 (82%) at corresponding timepoints (Figure 3C). Notably, 6-h phage exposure failed to induce statistically significant population declines (*p* value > 0.05) in both biofilm age groups, suggesting delayed lytic activity against embedded biofilms.

Extended phage exposure (12–24 h) demonstrated statistically significant reductions in both 72– and 96-h mature biofilms (*p* value < 0.05), as visualized in Figures 3C, D. Parallel trends emerged in comparative assessments against untreated biofilm controls (Figure 4), with CV staining analyses validating temporal reductions in biofilm biomass. Notably, no dose-dependent variability in biofilm eradication was observed between MOI-1 and MOI-0.1 at any time point (*p* value > 0.05). Furthermore, therapeutic duration (12 vs. 24 h) showed no statistically meaningful impact on biofilm clearance efficacy, regardless of biofilm maturation stage (*p* value > 0.05).

### 3.7 UHKP was stable at multiple environmental conditions

**Figure 5:**
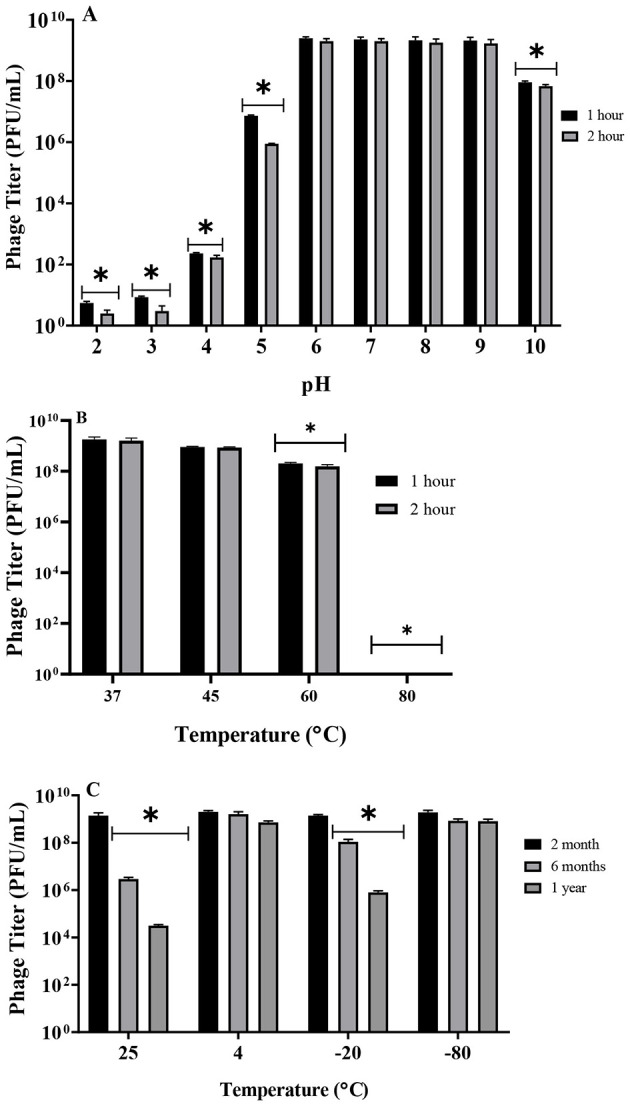
Stability of UHKP under different conditions. **(A)** Effect of pH treatments (1 and 2 h), **(B)** short-term effect of temperature (1 and 2 h) and **(C)** effect of long-term storage stability on UHKP viability. Mean titers from three independent experiments are shown as bar graphs, with error bars representing SEM. Asterisks indicate statistically significant differences compared with the corresponding untreated control at the same time point.

UHKP demonstrated robust stability across physiological conditions. The phage retained full infectivity (*p* value > 0.05) when exposed to pH 6–9 for 1–2 h. At pH 5 and 10, titers significantly decreased by 2 and 1 log (*p* value < 0.05), while pH 3 and 4 produced only few plaques and caused near-complete inactivation. Thermal stability testing revealed insignificant changes in titer at 37 and 45 °C, though 60 °C made statistically significant reduction by 1 log (*p* value < 0.05) and 80 °C completely inactivated UHKP. During storage, UHKP maintained stability at 4 °C,−20 °C, and −80 °C for 2 months (*p* value > 0.05). There was an insignificant change in titer by 2 month storage at all the tested temperatures (4, 25, −20, and −80 °C). After 6 months, significant reductions occurred at −20 °C (1 log) and 25 °C (2 logs) (*p* value < 0.05), while 4 °C and −80 °C resulted in no significant changes in titers through 1 year (*p* value > 0.05) (Figure 5).

### 3.8 UHKP has an extended latent period and short burst size

**Figure 6:**
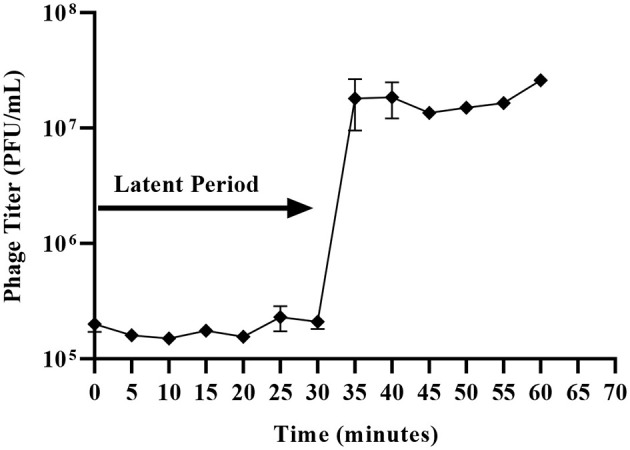
One-step growth curve of *Klebsiella* phage UHKP. A latent period of 30 min was observed, with an average burst size of 85 PFU per infected cell.

UHKP exhibited a latent period of 30 min, yielding an average burst size of 85 PFU per infected cell (Figure 6).

### 3.9 UHKP has a morphological resemblance to Podovirus

**Figure 7:**
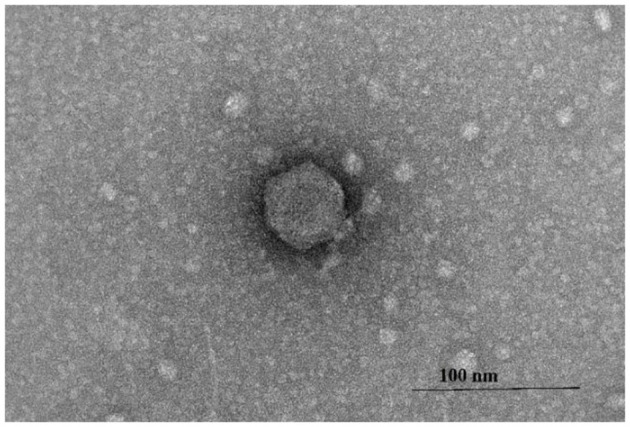
Transmission electron micrograph of UHKP, revealing icosahedral capsid with a short non-contractile tail similar to podovirus.

Transmission electron microscopy revealed that bacteriophage UHKP possesses an icosahedral head with an average diameter of approximately 56 ± 3 nm and a short, non-contractile tail measuring around 15 ± 2 nm in length (Figure 7). The virion exhibits the characteristic morphology of podovirus-like phages within the family *Podoviridae*, featuring a short tail structure directly attached to the capsid. This structural configuration is consistent with members of the genus *Lastavirus*, supporting the classification of UHKP as a lytic podophage. The clearly defined head symmetry and short tail appendage observed under TEM reinforce its identification as a podoviral phage infecting *Klebsiella* species.

### 3.10 Genome annotation

**Figure 8:**
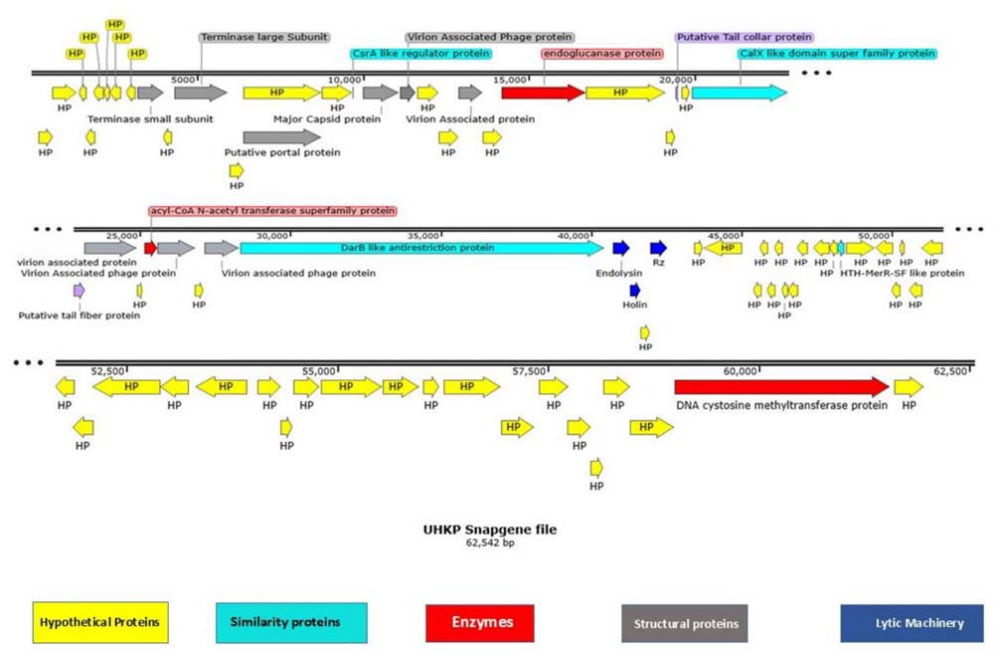
Linear Genome map of UHKP drawn with SnapGene 6.0.

The bacteriophage UHKP genome was analyzed for structural and functional annotation. The genome has a length of 62,542 base pairs (bp) with a GC content of 56.6%. GeneMarkS predicted 78 ORFs, while the RAST server predicted 82 ORFs, and Prokka by Galaxy predicted 77 ORFs. ORFs were considered coding sequences (CDS) only if they were longer than 120 bp and had a detectable Shine-Dalgarno sequence (SDS). After applying these criteria, a total of 77 ORFs were retained for further analysis. Of these, 22 ORFs (30%) encode proteins with known or predicted functions, while the remaining 55 ORFs (70%) are annotated as hypothetical proteins due to the absence of homologs in existing genomic databases. The largest gene in the genome is ORF 35, which spans 12,137 bp and encodes a DarB-like antirestriction protein. The smallest ORF is ORF 73, which is 157 bp long and encodes a hypothetical protein (Figure 8). The fully annotated genome sequence has been uploaded in the NCBI database under accession number PV287707.1.

Functional annotation revealed genes involved in DNA packaging, structural assembly, host lysis, and regulatory functions. The genome organization reflects a modular structure, with genes for DNA packaging and structural proteins clustered at the beginning and end of the genome, respectively, while regulatory and metabolic genes are scattered throughout. Structural proteins, such as the major capsid protein (ORF 16) and putative portal protein (ORF 13), are critical for phage structure. Additionally, genes encoding putative tail fiber (ORF 28) and tail collar proteins (ORF 25) were identified, which are likely involved in host recognition and attachment.

No Signal peptides were predicted in phage genes. Four transmembrane helices were identified, 2 in endoglucanase (ORF 22) and 2 in virion associated proteins (ORF 29), suggesting their involvement in membrane-associated functions such as host cell wall degradation and phage-host interaction. Analysis of the genome via PhagePromotor by Galaxy revealed putative promoter sequences and regulatory elements. The genome also contains several regulatory elements, including putative promoter sequences and Rho-independent terminators. These elements are likely involved in controlling gene expression during the phage life cycle.

### 3.11 Therapeutic potential and safety considerations of UHKP

The safety of bacteriophage UHKP for therapeutic applications was assessed through comprehensive genomic analysis. No integral prophage regions were detected in the UHKP genome using the PHASTER tool, indicating the absence of temperate or lysogenic elements. Both PhageAI and PHACTS confidently predicted UHKP to be a strictly lytic phage, further supporting its suitability for therapeutic use. The genome was screened for the presence of bacterial virulence factors, conjunctive and integrative elements, antibiotic resistance genes, and mycotoxins using multiple databases, and none were detected, confirming the absence of harmful genetic elements.

Additionally, no phage-encoded integrase or repressor genes were found in the UHKP genome, despite thorough annotation with multiple databases. This finding reinforces the lytic nature of UHKP and its inability to integrate into the host genome, which is a critical safety consideration for therapeutic phages. Host specificity analysis using the HostPhinder tool designated *Klebsiella pneumoniae* as the likely host for UHKP, based on genomic signatures and homology with known phage-host systems. This host specificity, combined with the absence of harmful genes and the confirmed lytic lifestyle, makes UHKP a promising candidate for therapeutic applications against *K. pneumoniae* infections.

The UHKP genome exhibited a pronounced codon bias, with arginine (CGC, 32.86%/1000) and glutamine (CAG, 29.55/1000) triplets demonstrating the highest usage frequencies, while threonine encoding codons showed minimal utilization (18.5%). Computational screening using ARAGORN and tRNAScan-SE 2.0 confirmed the absence of tRNA or rRNA genes in UHKP genome. Genomic characterization of UHKP via RepeatMasker revealed no long terminal repeats (LTRs), one long interspersed nuclear element (LINE), short interspersed nuclear elements (SINEs), and one DNA transposon. Five simple repetitive sequences were additionally annotated. Tandem Repeat Finder further identified a 21-nucleotide motif recurring at 2.0 copies. CRISPR-Cas systems were absent in the UHKP genome.

### 3.12 . Phage UHKP encodes a comprehensive suite of enzymes for DNA metabolism and replication

For DNA replication and repair, UHKP utilizes a DNA cytosine methyltransferase (ORF 76) that may play roles in host evasion and gene regulation, along with the DarB-like antirestriction protein (ORF 35) that counters host restriction-modification systems (Tock and Dryden, 2005; Oliveira et al., 2014). The genome also encodes terminase small subunit (ORF 9) and large subunit (ORF 11) for DNA packaging during virion assembly (Lokareddy et al., 2022). For transcriptional regulation, UHKP possesses a CsrA-like regulator protein (ORF 15) that may modulate post-transcriptional processes (Gorelik et al., 2024), and the HTH-MerR-SF like protein (ORF 51) likely functions as a transcriptional regulator (Brown et al., 2003). These annotations indicate that UHKP genome encodes multiple proteins associated with DNA replication, packaging, and transcriptional regulation.

### 3.13 UHKP features a well-defined three-step lysis cassette for host cell disruption

Endolysin (ORF 36), holin (ORF 37), and Rz protein (ORF 39) work in concert to mediate efficient host lysis. These ORFs show precise genomic organization with minimal overlap. Both endolysin and holin contain predicted signal peptides and transmembrane helices, indicating their membrane-associated functions. The endolysin of UHKP belongs to the lysozyme superfamily and likely contains a single catalytic domain for peptidoglycan degradation. The holin protein (ORF 37) features a characteristic N-terminal transmembrane domain and a charged C-terminus, typical of class II holins. The Rz protein (ORF 39) completes the lysis system by disrupting the outer membrane, with structural predictions suggesting it contains a single transmembrane helix. This coordinated lysis system demonstrates UHKP's efficient strategy for host cell exit and virion release. Notably, the genome encodes an endoglucanase protein (ORF 22), that can degrade polysaccharides and may assist the phage in breaching bacterial biofilms or cell walls during infection, suggesting a specialized adaptation that enhances phage infectivity or spread within certain bacterial communities.

### 3.14 UHKP belongs to the genus *Lastavirus* within the class *Caudoviricetes*

**Figure 9:**
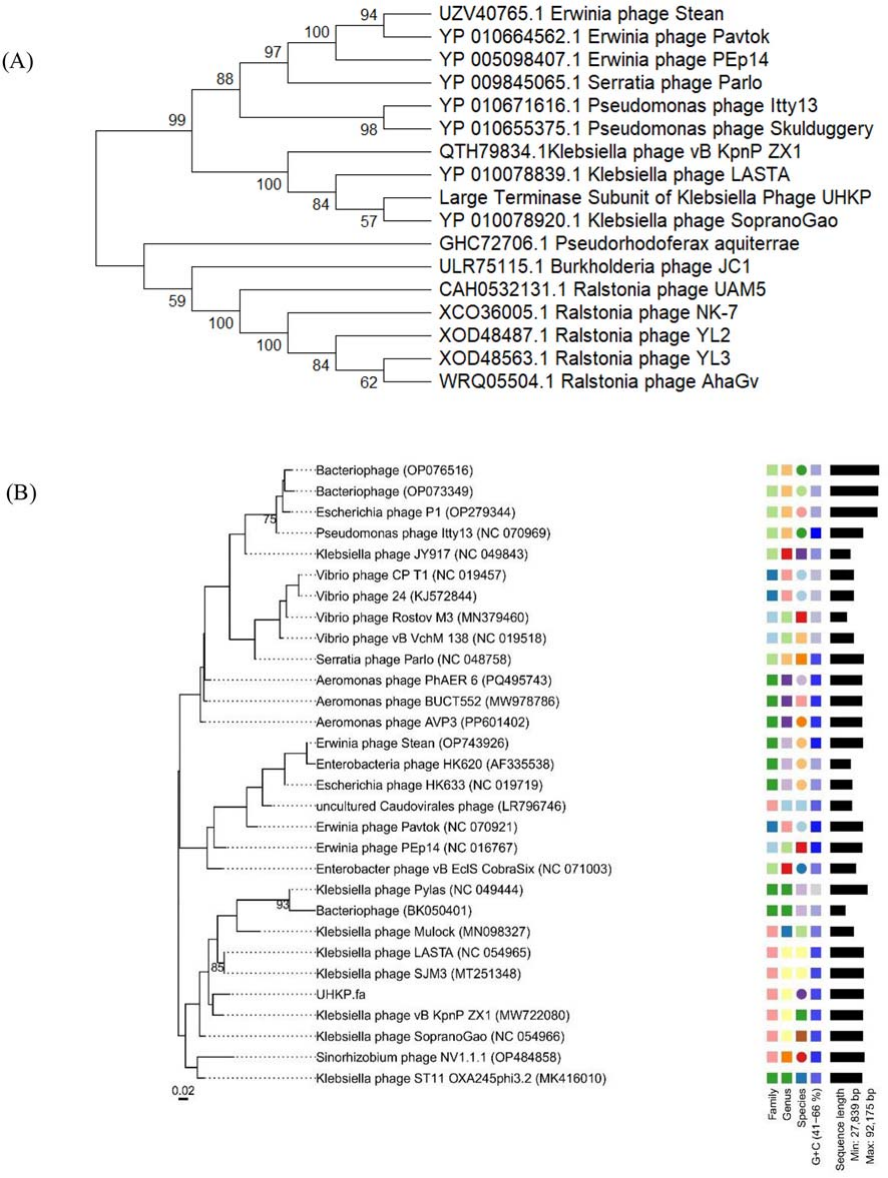
Phylogenetic tree of the large terminase subunit **(A)** and whole-genome sequences **(B)** of UHKP constructed using MEGA X and VICTOR. The UPGMA method with 1,000 bootstrap replicates was applied for tree construction in MEGA X while the default setting of VICTOR was used to construct a whole-genome tree.

**Figure 10:**
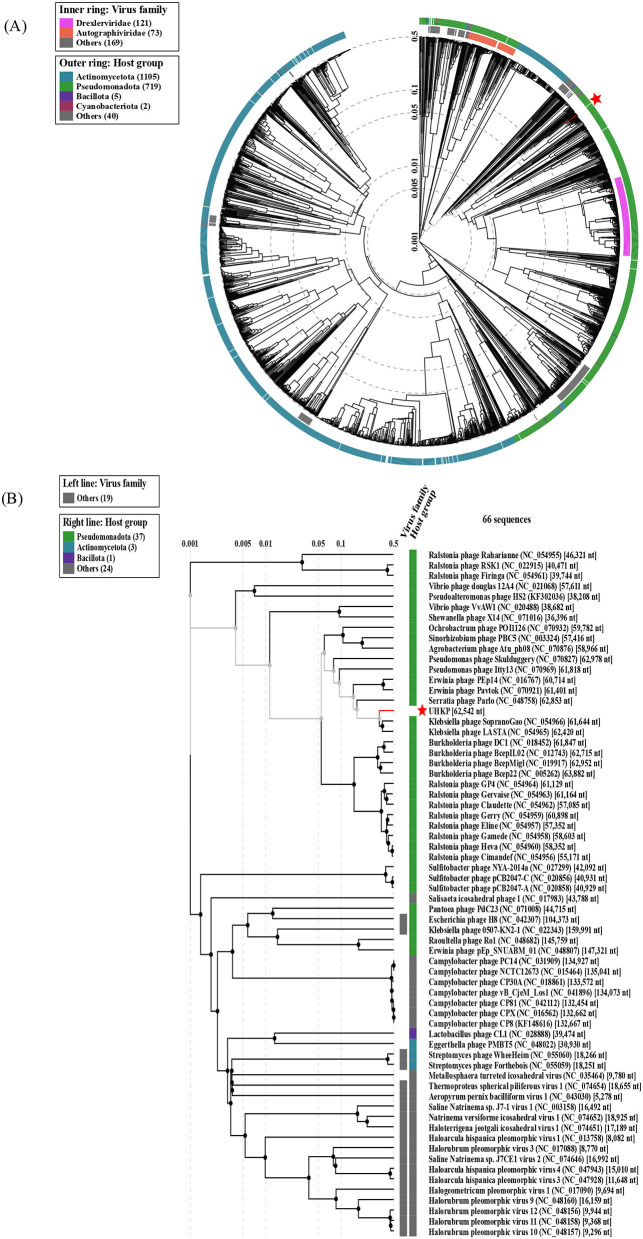
Proteomic tree constructed using VIPTree of phage UHKP **(A)** Circular tree of related phages of RefSeq genomes and top BLASTn hits, **(B)** Rectangular tree of phage UHKP representing subset of closely related phages from circular tree.

According to BLASTn and ANI calculations, the closest homologs of UHKP are *Klebsiella* phages SopranoGao, vB_KpnP_ZX1, SJM3, and LASTA, sharing 94–95% nucleotide identity with 67–74% query coverage. These phages, along with UHKP, are classified within the class *Caudoviricetes*, specifically under the genus *Lastavirus* in the viral realm *Duplodnaviria*. UHKP possesses a 62,542 bp genome, encoding 77 predicted ORFs with a GC content of 56.6%, which is consistent with other members of this lineage. The phylogenetic tree of UHKP's large terminase subunit (TerL) reveals its close evolutionary relationship with other *Klebsiella* phages, including vB_KpnP_ZX1 (MW722080), LASTA (NC_054965), and SopranoGao (NC_054966), forming a distinct clade separate from phages infecting other genera (Figure 9A). The VICTOR nucleotide tree place UHKP within a monophyletic clade of *Klebsiella* phages, including vB_KpnP_ZX1, LASTA, and SopranoGao, suggesting shared evolutionary ancestry. Notably, UHKP clusters distantly from *Escherichia* phages (e.g., HK620, P1) and *Vibrio* phages (e.g., CP T1, Rostov M3), reflecting host-specific divergence (Figure 9B). Whole-proteome–based phylogenetic analysis of bacteriophage UHKP was performed using the VIPTree server. The generated tree positioned UHKP within a well-supported monophyletic cluster of *Klebsiella*-infecting podophages belonging to the genus *Lastavirus* under the class *Caudoviricetes*. UHKP exhibited closest proteomic relatedness to *Klebsiella phages* LASTA, SopranoGao, and vB_KpnP_ZX1, corroborating the results obtained from BLASTn, ANI, and terminase-based phylogenetic analyses (Figure 10).

## 4 Discussion

The *Klebsiella* phage UHKP was isolated using a multidrug-resistant *K. pneumoniae* host (strain *KP-*03). UHKP produced clear plaques when infected *KP-*03 and other strains whereas double zones were observed on *KP-*8890 bacterial lawn with expanding hazy halo after prolonged incubation (Figure 1). The double-zone observed specifically on strain *KP-*8890 is most likely due to capsule-specific depolymerase activity encoded by phage UHKP. *KP-*8890 is a confirmed K-17 serotype, whose capsular polysaccharide composition differs from that of other susceptible strain (Asif et al., 2023). Depolymerases act in a host-capsule–dependent manner, producing halos only when the enzyme efficiently degrades the specific capsule type. This phenomenon has been widely reported for *Klebsiella* phages exhibiting serotype-restricted depolymerase activity (Lukianova et al., 2023). These features mirror those of other *Klebsiella* phages encoding depolymerases (Lin et al., 2014; Lu et al., 2019).

UHKP exhibited a highly restricted host range. In spot assays it infected only a few clinical *K. pneumoniae* isolates (including strain *KP-*03, *KP-*05, *KP-*08, *KP-*11, and a K-17 serotype strain *KP-*8890) (Figure 1C) and showed no activity against other serotypes or non-*Klebsiella* species. This narrow specificity is typical for *Klebsiella* phages: for example, phage K5 lysed only its K21-type host (Lukianova et al., 2023), and a K1-specific phage infected all K1 strains but none of other capsular types (Lin et al., 2014). In practice, narrow host range can be advantageous for targeted therapy, though it limits coverage of diverse strains. Notably, UHKP's host *KP-*03 was clinically MDR, highlighting the phage's potential against resistant infections.

*In-vitro* assays showed that UHKP suppresses planktonic *K. pneumoniae* in a dose-dependent manner. At an MOI of 1, bacterial growth was stopped by 4 h and cultures were completely cleared by 8 h. In contrast, the MOI 0.1 delayed lysis and a reduction was seen only after 6 h and small amounts of growth persisted. These classic one-step kinetics (lag phase then rapid decline) are typical of lytic phages (Figure 2).

In this study, biofilm reduction was evaluated using a baseline-referenced approach, in which biofilm biomass measured at the 0-h time point served as the experimental control. This strategy follows established phage–biofilm methodologies and allows direct assessment of phage-mediated biofilm disruption relative to the pre-treatment state rather than comparison with parallel untreated time-matched controls, as described by Abedon et al. (2021). When analyzed relative to this baseline, UHKP produced marked reductions in young biofilms (24–48 h), consistent with our stated hypothesis. In contrast, reduced effects were observed against mature biofilms (72–96 h), which likely reflects increased structural complexity and tolerance of established biofilms, as reported in the broader phage–biofilm literature (Mayorga-Ramos et al., 2024). Treatment at MOI 1 removed 65%, 93% and 98% of 24-h-old biofilm after 6, 12, and 24 h, respectively (vs. untreated controls). Likewise, 48-h biofilms were reduced by 53%, 85% and 96% at 6, 12, and 24 h. In fact, after 24 h UHKP eradicated >98% of 24-h and >96% of 48-h biofilms, similar to JKP2′s reported 98% (24-h) and 96% (48-h) reductions (Asif et al., 2023). However, UHKP was less effective on older biofilms: only 50–87% of 72-h biofilm and 46–82% of 96-h biofilm were cleared by 24 h. In particular, 6-h treatments did not significantly reduce 72-h or 96-h biofilms (p>0.05), while 12–24 h exposure produced significant but incomplete clearance (Figure 3C). Thus, as biofilms matured, UHKP's activity dropped, consistent with many phage studies (Townsend et al., 2020) where dense, polysaccharide-rich matrices delay phage penetration. No significant difference was observed between MOI 1 and 0.1 for biofilm killing at any time point, indicating that even low phage doses gradually erode the biofilm given sufficient time. Similarly, extending the treatment from 12 to 24 h produced no further significant gains in removal, suggesting that UHKP's biofilm-penetrating ability reaches a limit without additional interventions. This finding refines previous recommendations for phage therapy duration (Domingo-Calap et al., 2020) by establishing age-dependent treatment windows. The persistent residual burden (104-107 CFU) observed across all biofilm ages confirms a fundamental limitation of phage monotherapy that has been consistently reported (Yang et al., 2020), strongly supporting the need for optimized combination approaches. In practice, combining phages with antibiotics or matrix-degrading enzymes often boost biofilm clearance. Phage–antibiotic synergy has been documented against *K. pneumoniae* (phage plus gentamicin greatly improved killing) (Guo et al., 2025), and phage-encoded enzymes can degrade EPS to expose cells (Duerkop et al., 2016). Therefore, UHKP's biofilm disruption may be enhanced by such combinatorial treatments in future studies. The presence of UHKP's plaque halo and its putative depolymerase might already aid EPS degradation, but auxiliary agents could further improve removal of mature biofilms.

Physiochemical characterization revealed UHKP's stability across a broad pH range (6–9) and at physiological temperatures (37 °C), though extreme pH (< 5, >10) and heat (>60 °C) caused significant titer loss. Storage at 4 °C and −80 °C maintained viability for 12 months, whereas −20 °C and room temperature led to progressive inactivation (Figure 5C). The physicochemical properties of UHKP show important similarities and distinctions when compared to other well-characterized *K. pneumoniae* phages. UHKP's stability across pH 6–9 matches the pH tolerance range reported for *Klebsiella* phage K5-4 (Hsieh et al., 2017), though UHKP demonstrates superior thermal resilience at 60 °C where K5-4 showed complete inactivation (Figures 5A, B). This enhanced stability could prove advantageous for clinical formulations, particularly for topical applications where environmental fluctuations may occur. Storage stability results align with findings for *Klebsiella* phage KpV74 (Volozhantsev et al., 2022), where −80 °C preservation maintained viability for >12 months, while the observed titer loss at −20 °C corroborates their warning against conventional freezer storage for therapeutic phages. Notably, UHKP's room temperature stability exceeds that of *Klebsiella* phage KpJH46Φ2 (Cano et al., 2021), which lost 3-log viability after 30 days at 25 °C compared to UHKP's more gradual decline.

UHKP exhibited a latent period of 30 min, followed by a lytic burst phase initiating between 45–50 min post-infection, yielding an average burst size of 85 PFU/cell (Figure 6). UHKP's replication kinetics (30-min latent period, 85 PFU/cell burst size) align with recent findings for *Klebsiella* podoviruses but reveal host-specific adaptations. Phage KpJH46Φ2 (Cano et al., 2021) exhibits similar latent period (30 min) and burst size (60 PFU/cell), and myophage KpM9 (Chen et al., 2023) shows larger bursts (120 PFU/cell) with longer delays (60 min). UHKP strikes an intermediate balance, similar to the newly characterized *Klebsiella* podovirus *KP-*8E1 (Wang et al., 2024), which shares both extended latent phases (40–50 min) and moderate burst sizes (65–80 PFU/cell).

The UHKP genome (62,542 bp, 56.6% GC content) exhibits characteristic features of *Klebsiella*-infecting podoviruses but with distinct evolutionary signatures while similar in size to *K. pneumoniae* phages KpV74 (44,094 bp) and Kp34 (43,809 bp) (Lu et al., 2019; Cano et al., 2021). UHKP's higher GC content (56.6% vs. 50.2-54% in most *Klebsiella* podoviruses) suggests potential host adaptation to clinical MDR strains, which often show elevated GC content in virulence loci (Rodrigues et al., 2022). The 77 predicted ORFs include conserved structural modules seen in other *Klebsiella* phages. UHKP's major capsid protein (ORF16) shares 82% aa identity with *K. pneumoniae* phage KpJH46Φ2 (Cano et al., 2021). The putative tail fiber protein (ORF28) contains depolymerase domains homologous to those in phage K5-2 (Hsieh et al., 2017), explaining observed plaque formation. The holin-endolysin-Rz cassette shows 78% synteny with *K. pneumoniae* phage KpV74 (Volozhantsev et al., 2022), but with a 12-aa insertion in the endolysin catalytic domain.

Phylogenetically, UHKP clusters with the *Drulisvirus* genus, closely related to *K. pneumoniae* phages KpJH46Φ2 and Kp34. Like these phages, UHKP uses direct terminal repeats (DTRs) for packaging, a feature shared by 89% of sequenced *Klebsiella* podoviruses (Gomez-Ochoa et al., 2022). The absence of tRNA genes aligns with 92% of *Klebsiella* podoviruses (NCBI database), while the lack of CRISPR elements contrasts with *K. pneumoniae* phage Kp1, which encodes a functional CRISPR array (Kim et al., 2023). Most significantly, UHKP shows no mobile genetic elements (e.g., integrases, transposases), a critical safety advantage over temperate *Klebsiella* phages (Figure 8). As per the updated International Committee on Taxonomy of Viruses (ICTV) classification framework, *Klebsiella virus* UHKP has been classified as an unassigned species within the *Drulisvirus* genus of the *Autographiviridae* family. Genomic analysis reveals >90% nucleotide identity with *Drulisvirus* members *Klebsiella* phage *SopranoGao, vB_KpnP_ZX1, SMJ3*, and *LASTA*. The phage's genomic architecture aligns closely with its taxonomic relatives, exhibiting a GC content of 56.6% and a genome size consistent with other *Drulisvirus* phages.

ORFs 36, 37, and 39 of the UHKP phage comprise a canonical three-component lysis system, functioning synergistically to ensure timely and effective host cell lysis. The genomic organization of these lysis genes is precise, with minimal intergenic overlap, a structural arrangement reminiscent of well-characterized lytic phages such as T7 (Cahill and Young, 2019). UHKP's endolysin (ORF 36), a member of the lysozyme superfamily, is predicted to contain a single catalytic domain, most likely an N-acetylmuramidase, for targeted peptidoglycan degradation which is a common enzymatic function among phage-derived lysins (Schmelcher et al., 2012; Rodríguez-Rubio et al., 2013). The presence of a signal peptide and transmembrane helix in the endolysin supports a secretion mechanism similar to the SAR (signal-anchor-release) endolysins seen in Pseudomonas phage ϕKMV and coliphage P21 (Briers et al., 2007; Sun et al., 2009). ORF 37 encodes a holin with a distinct N-terminal transmembrane helix and charged C-terminal domain, classifying it as a class II holin known for controlling the timing of endolysin release via membrane permeabilization (Young, 2014; Kongari et al., 2018). Structural predictions for ORF 39 indicate a single transmembrane helix typical of spanin components, aligning with the function of Rz-like proteins in disrupting the outer membrane during the final stage of host cell lysis (Catalao et al., 2013). The coordinated presence of SAR-endolysin, class II holin, and Rz-spanin suggests that UHKP employs a temporally regulated, efficient lysis mechanism, paralleling strategies observed in other *Klebsiella* phages like JKP2 and Phage 117 (Tan et al., 2019; Asif et al., 2023). These findings underscore the evolutionary conservation and mechanistic efficiency of phage lysis modules tailored for rapid host exit and progeny dissemination.

UHKP's structural module is typical of podoviruses, consistent with its assignment to the Drulisvirus genus. The genome's structural genes are organized in a conserved head-tail cassette. ORFs 16–18 encode the major capsid protein and internal core proteins, analogous to the head assembly proteins of other *Klebsiella* podophages (Asif et al., 2023). Notably, ORF28 is predicted to encode a tail fiber with a polysaccharide-degrading domain, where tail fibers or spikes bearing capsule depolymerases are a common adaptation in *Klebsiella* phages for capsular recognition and breakdown (Wu et al., 2023). Similarly, ORF19 encodes a tail tubular protein (TTPA homolog) with high similarity to those in phages like KpV74 and KP34, reflecting the deep conservation of the tail assembly module among Drulisviruses (Lu et al., 2019; Volozhantsev et al., 2022). Phylogenetic analysis of the large terminase subunit (ORF11) and whole genome sequences (Figures 9A, B) places UHKP within the *Lastavirus* clade, clustering with phages vB_KpnP_ZX1, LASTA, and SopranoGao, which share high genomic identity and evolutionary ancestry. Proteomic phylogenetic analysis generated using VIPTree (Figure 9B) positioned bacteriophage UHKP within the genus *Lastavirus* under the family Drexlerviridae, realm Duplodnaviria. The phage clustered tightly with members infecting *Klebsiella* hosts, including *vB_KpnP_ZX1, LASTA*, and *SopranoGao*, indicating strong proteomic homology and shared evolutionary ancestry.

Genome analysis of UHKP revealed no identifiable virulence factors, antibiotic resistance genes, or lysogenic elements, suggesting that it lacks obvious genetic features contraindicating therapeutic evaluation. However, conclusions regarding therapeutic safety cannot be made based solely on genomic analysis. As noted in previous systematic reviews, phage therapy can be associated with endotoxin release due to bacterial lysis and variable immune responses, and adverse events, although generally rare, have been reported in animal and human studies (Liu et al., 2021). Structured safety assessments, including evaluation of host responses, interactions with human microbiota, and potential inflammatory reactions, are necessary before proposing clinical application.

## 5 Conclusion

UHKP is a newly characterized lytic phage against MDR *K. pneumoniae*, producing a depolymerase-typical plaque halo. It has a very narrow host range (including K-17 serotype), effectively kills planktonic bacteria, and significantly disrupts early biofilms. Its activity against mature biofilms is moderate with similar phages. UHKP is stable at physiological pH/temperature and can be stored long-term at 4 °C or −80 °C. The genome confirms a strict lytic, phage therapy-friendly profile. UHKP produced diminished but statistically significant reductions against very mature biofilms. These patterns, rapid and near-complete disruption of early biofilms, reduced penetration into dense, aged matrices, and a clear dependence on exposure time are internally consistent across duplicate assays and emphasize that phage-mediated matrix destabilization and host lysis are effective but can be constrained by EPS architecture in mature biofilms. Taking together, these results establish UHKP as a promising candidate for phage therapy against *K. pneumoniae* and data therefore supports phage use for early or device-associated biofilm control and imply that for entrenched biofilms combination approaches (phage cocktails, enzymatic depolymerases or adjunctive antibiotics) may be required.

## Data Availability

The datasets presented in this study can be found in online repositories. The names of the repository/repositories and accession number(s) can be found at: https://www.ncbi.nlm.nih.gov/PV287707.1.
