# Supplementary material for: Genomic and functional characterization of a lytic Klebsiella phage UHKP with antibiofilm activity
Source: Front Microbiol. 2026 Mar 2;17:1775638. doi: 10.3389/fmicb.2026.1775638 (PMC12989515; doi:10.3389/fmicb.2026.1775638)
Supplement: Supplementary file 1 [file Table_1.docx]

| **Strain #** | **Isolation source** | **AK** | **CN** | **TOB** | **CIP** | **LEV** | **IMP** | **MEM** | **CAZ** | **FEP** | **TZP** | **AZT** | **PB** | **CT** |
| --- | --- | --- | --- | --- | --- | --- | --- | --- | --- | --- | --- | --- | --- | --- |
| KP-01 | Urine | R | R | S | R | R | S | S | R | R | R | R | S | S |
| KP-02 | Wound swab | R | R | R | R | R | S | S | R | R | S | S | S | S |
| **KP-03** | Blood | R | R | R | R | R | R | R | R | R | R | R | S | S |
| KP-04 | Pus | R | S | S | R | R | S | S | R | R | R | R | S | S |
| KP-05 | Urine | S | S | R | R | R | S | S | S | S | S | R | S | S |
| KP-06 | Wound swab | R | R | S | S | S | S | R | S | R | R | S | S | S |
| KP-07 | Urine | R | R | R | S | S | R | S | S | S | R | R | S | S |
| KP-08 | Pus | R | S | R | R | S | R | R | R | R | R | R | S | S |
| KP-09 | Blood | R | R | S | S | R | S | R | R | R | S | S | S | S |
| KP-10 | Urine | S | S | S | R | R | S | S | S | R | R | R | S | S |
| KP-11 | Wound swab | R | S | R | R | R | S | R | S | S | S | R | S | S |
| KP-12 | Pus | R | R | R | R | R | R | R | R | R | R | R | S | S |
| KP-13 | Urine | S | S | R | S | S | R | S | R | R | R | R | S | S |
| KP-14 | Urine | R | S | S | R | R | R | R | R | R | R | R | S | S |
| KP-15 | Pus | R | R | R | R | R | S | S | R | S | R | S | S | S |
| KP-16 | Pus | R | R | S | R | S | R | S | S | R | S | S | S | S |
| KP-17 | Pus | R | R | R | S | S | S | R | S | R | S | S | S | S |
| KP-18 | Blood | S | S | S | S | S | S | S | R | S | R | S | S | S |
| KP-19 | Tissue | S | R | S | R | R | R | S | R | R | R | R | S | S |

**Supplementary Table S1:** Antimicrobial susceptibility profile of *Klebsiella pneumoniae* clinical isolates determined by Kirby–Bauer disk diffusion assay

**Abbreviations:**

AK: Amikacin, CN: Gentamicin, TOB: Tobramycin, CIP: Ciprofloxacin, LEV: Levofloxacin,
IMP: Imipenem, MEM: Meropenem, CAZ: Ceftazidime, FEP: Cefepime,
TZP: Piperacillin–Tazobactam, AZT: Aztreonam, PB: Polymyxin B, CT: Colistin
